# First report of *Eucoleus boehmi* (syn. *Capillaria boehmi*) in dogs in north-western Italy, with scanning electron microscopy of the eggs

**DOI:** 10.1051/parasite/2012194433

**Published:** 2012-11-15

**Authors:** M. Magi, L. Guardone, M.C. Prati, B. Torracca, F. Macchioni

**Affiliations:** 1 Dipartimento di Patologia Animale Profilassi e Igiene degli Alimenti, Università di Pisa Viale delle Piagge 2 56127 Pisa Italia; 2 Scuola Normale Superiore di Pisa Piazza dei Cavalieri 7 56126 Pisa Italia

**Keywords:** *Eucoleus boehmi*, *Eucoleus aerophilus*, *Trichuris vulpis*, dog, Italy, SEM, *Eucoleus boehmi*, *Eucoleus aerophilus*, *Trichuris vulpis*, chien, Italie, MEB

## Abstract

Dogs can be infected by several nematodes of the Trichuridae family. Trichuridae eggs are all similar, barrel shaped with polar plugs, and misdiagnosis among different species can occur. The most common species is *Trichuris vulpis*, while the respiratory parasites *Eucoleus boehmi* (syn. *Capillaria boehmi*) and *Eucoleus aerophilus* (syn. *Capillaria aerophila*) are rarely observed in pets. *E. boehmi* is reported for the first time in this study in north-western Italy with other Trichuridae. Dog faecal samples (270) were examined by flotation. *E. boehmi* (2.2%), *E. aerophilus* (4.4%) and *T. vulpis* (12.2%) were found; identification was done with measurements and through observation of morphological characters already known. The specific identification of *E. boehmi* was confirmed using scanning electron microscopy: its egg shell shows a dense network with a fine mesh, surrounding small pits, on the contrary *E. aerophilus* eggs present a thick mesh with wide depressions, while *T. vulpis* eggs surface is smooth.

## Abstract (fr)

Les chiens peuvent être infectés par plusieurs nématodes de la famille des Trichuridae. Les oeufs de Trichuridae sont tous en forme de tonneau avec des bouchons polaires, et un diagnostic erroné parmi les différentes espèces peut survenir. L’espèce la plus commune est *Trichuris vulpis*, tandis que les parasites des voies respiratoires, *Eucoleus boehmi* (syn. *Capillaria boehmi*) et *Eucoleus aerophilus* (syn. *Capillaria aerophila*), ne sont guère observés chez les animaux de compagnie. La présence de *E. boehmi* est rapportée pour la première fois dans cette étude dans le nord-ouest de l’Italie avec les autres Trichuridae. Des échantillons fécaux de chiens (270) ont été examinés par flottation. Les prévalences étaient de 2,2 % pour *E. boehmi*, de 4,4 % pour *E. aerophilus* et de 12,2 % pour *T. vulpis*. Les oeufs ont été identifiés par les mensurations et l’observation des caractères morphologiques déjà connus. L’identification spécifique de *E. boehmi* a été confirmée par microscopie électronique à balayage : la coque de l’oeuf a un réseau dense avec une maille fine, qui entoure de petites fosses, alors que les oeufs de *E. aerophilus* présentent une maille épaisse avec des dépressions larges, tandis que la surface des oeufs de *T. vulpis* est lisse.

Dogs can be infected by several nematodes belonging to the Trichuridae family. The most common species is the intestinal whipworm *Trichuris vulpis* (Frolich, 1789), while the respiratory parasites *Eucoleus boehmi* (syn. *Capillaria boehmi*) (Supperer, 1953) and *Eucoleus aerophilus* (syn. *Capillaria aerophila*) (Creplin, 1839) are rarely reported in pets.

All Trichuridae eggs are characterized by a similar barrel shape with polar plugs and misdiagnosis among different species can occur. *E. boehmi* eggs are 54- 60 × 30-35 µm in size, contain a multicellular embryo that does not fill the egg, are clear to golden in colour and show a delicately pitted surface (Campbell, 1991; Schoning *et al.*, 1993; Zarnowsky & Patyk, 1960). *E. aerophilus* eggs measure 60-72 × 26-34 µm, are entirely filled by one- or two-cell embryo, have a net-like ornamented outer layer and are slightly asymmetrical. *T. vulpis* eggs measure 70-80 × 30-42 µm, have a brown-yellowish colour, a symmetrical shape with prominent plugs showing a ring-like thickening at the base and a smooth surface (Campbell, 1991; Conboy, 2009). Respiratory capillariosis in pets has recently received increasing attention in Europe and North America, although specific coprological diagnosis can be challenging (Conboy, 2009; Di Cesare *et al.*, 2011).

The aim of this work is to report for the first time the observation of *E. boehmi* in dogs in north-western Italy, together with other Trichuridae, and to describe the egg surface with scanning electron microscopy, comparing with *E. aerophilus* and *T. vulpis* eggs.

## Materials and Methods

From January 2010 to March 2011, as part of a wider study on canine endoparasites, 270 faecal samples of dogs living in a rural environment were randomly sampled in Liguria, north-western Italy. The examined population consisted of 161 male dogs and 109 females (age range: 6 months-14 years; average: 5.4 years). Samples were examined by centrifugal flotation with zinc sulphate (s.g. 1.350).

Trichuridae eggs were identified on the basis of morphologic and morphometric characteristics described in the literature (Campbell, 1991; Conboy, 2009; Schoning *et al.*, 1993). *E. boehmi* eggs were also examined using scanning electron microscopy (JEOL JSM 5410 SEM). For SEM, eggs were isolated by flotation in a zinc sulphate solution and sieved, following the technique described in Al-Sabi *et al.* (2010), then mounted on aluminum stubs, air dried, gold coated with the sputtering technique, observed and photographed.

Sterile cotton tip swabs were inserted into each nasal passage for nasal examination, following the technique described by Schoning *et al.* (1993).

## Results

**Fig. 1.**
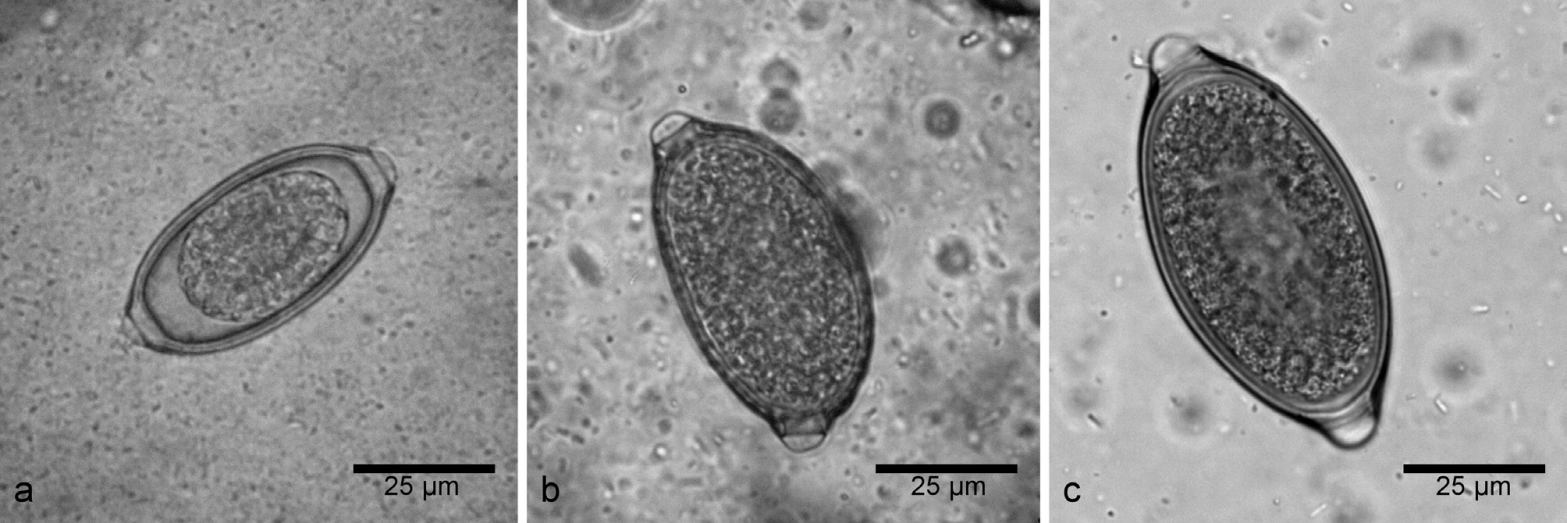
Light microscopy: a. *Eucoleus boehmi* egg; b. *Eucoleus aerophilus* egg; c. *Trichuris vulpis* egg.

**Fig. 2.**
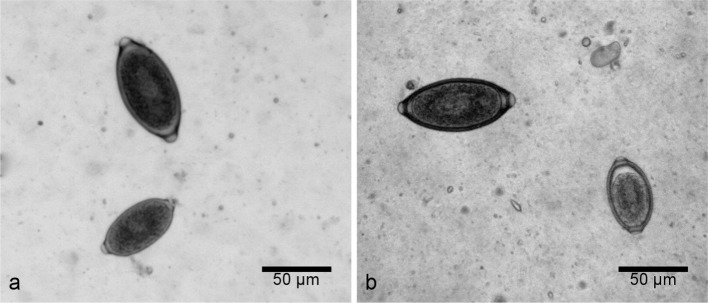
Light microscopy: a. *Eucoleus aerophilus* egg (bottom) and *Trichuris vulpis* egg (top); b. *Eucoleus boehmi* egg (right) and *Trichuris vulpis* egg (left).

Of the 270 examined faecal samples, 46 were positive for Trichuridae eggs: six dogs (2.2 %) were positive for *E. boehmi* (Fig. 1a), 12 (4.4 %) for *E. aerophilus* (Fig. 1b), and 33 (12.2 %) for *T. vulpis* (Fig. 1c). Five dogs presented a double infection (three cases of *T. vulpis* and *E. aerophilus*, Fig. 2a, two cases of *T. vulpis* and *E. boehmi*, Fig. 2b). Nasal swabs performed on all six dogs positive for *E. boehmi* were negative.An accurate morphological and morphometric analysis was carried out using the optical microscope to identify eggs of different species, following the already mentioned characteristics (Campbell, 1991; Conboy, 2009; Schoning *et al.*, 1993).

**Fig. 3.**
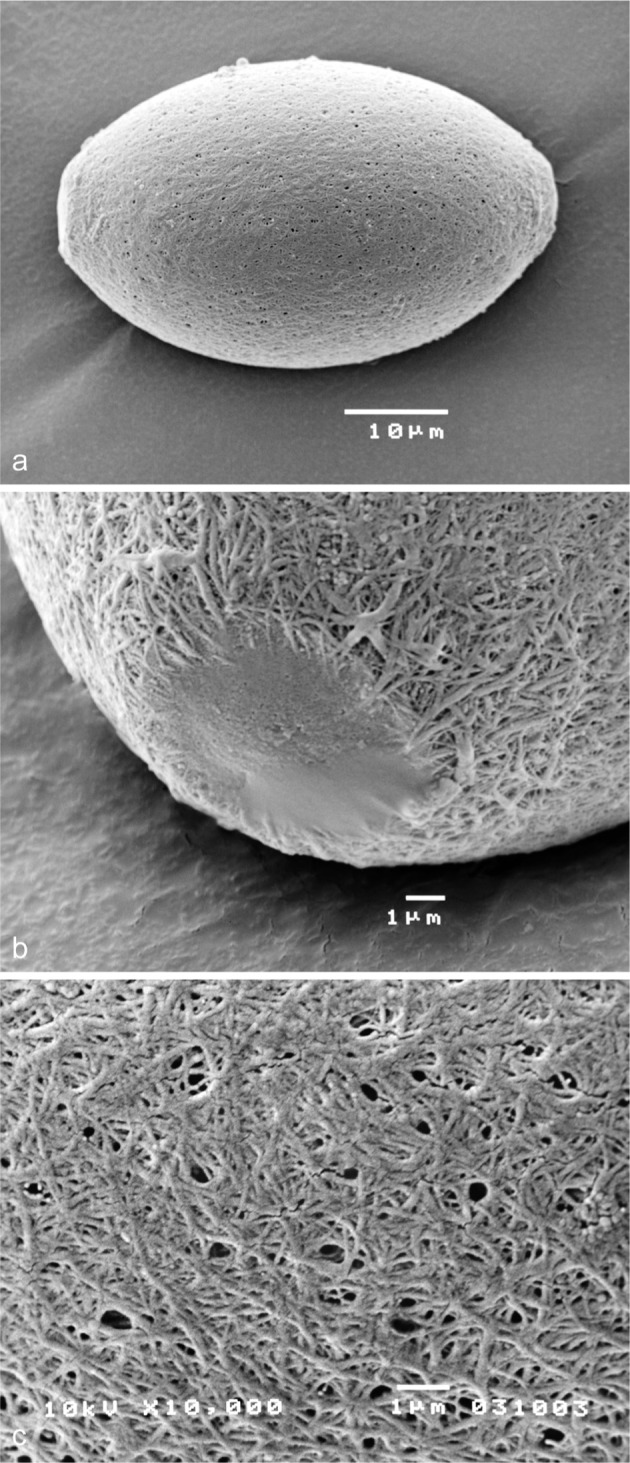
Scanning electron microscopy of *Eucoleus boehmi* egg: a. whole egg; b. polar plug; c. egg surface.

Scanning electron microscopy highlighted the characteristics of the *E. boehmi* egg shell: the surface presented a dense network with a fine mesh, surrounding irregularly distributed small pits, which give the eggs a porous appearance (Fig. 3). These features clearly distinguish *E. boehmi* eggs from *E. aerophilus* and *T. vulpis*. With SEM, *E. aerophilus* eggs present a thick mesh with wide depressions, while *T. vulpis* eggs surface is completely smooth (Traversa *et al.*, 2011).

In pet animals, respiratory capillariosis are likely to be underestimated because of difficulties in coprologic specific diagnosis. The confusion arises especially from the similarity of the barrel-shape of the Trichuridae eggs. The most reliable distinctive characteristic is the external structure of the shell (Zarnowsky & Patyk, 1960; Conboy, 2009). This feature can already be observed with an accurate morphological analysis of the egg surface using light microscopy (100 ×); scanning electron microscopy highlighted in details the characteristics of the egg shell structure which differentiate the three species.

Concerning the clinical aspects, only one dog infected in our study showed mild respiratory symptoms, such as nasal discharge and cough during exercise.

Although respiratory capillariasis is considered uncommon, clinicians should include these infections in the differential diagnosis of cardiopulmonary affections. Clinicians should thus consider performing accurate coprological examinations in the case of respiratory symptoms, using more sensitive solutions for flotation (zinc sulphate) (Conboy, 2009). Furthermore, the awareness of the possible presence of different species of capillarid nematodes in dogs is essential for a correct diagnosis. Epidemiological studies are required to ascertain the actual distribution of these neglected and emerging parasites.
